# Enhancing translational team effectiveness: The Wisconsin Interventions in Team Science framework for translating empirically informed strategies into evidence-based interventions

**DOI:** 10.1017/cts.2021.825

**Published:** 2021-07-21

**Authors:** Betsy Rolland, Sarah D. Hohl, LaKaija J. Johnson

**Affiliations:** 1 Institute for Clinical and Translational Research, School of Medicine and Public Health, University of Wisconsin-Madison, Madison, WI, USA; 2 Carbone Cancer Center, School of Medicine and Public Health, University of Wisconsin-Madison, Madison, WI, USA

**Keywords:** Framework, team science, translational teams

## Abstract

Achieving the clinical, public health, economic, and policy benefits of translational science requires the integration and application of findings across biomedical, clinical, and behavioral science and health policy, and thus, collaboration across experts in these areas. To do so, translational teams need the skills, knowledge, and attitudes to mitigate challenges and build on strengths of cross-disciplinary collaboration. Though these competencies are not innate to teams, they can be built through the implementation of effective strategies and interventions. The Science of Team Science (SciTS) has contributed robust theories and evidence of empirically-informed strategies and best practices to enhance collaboration. Yet the field lacks methodological approaches to rigorously translate those strategies into evidence-based interventions to improve collaborative translational research. Here, we apply lessons from Implementation Science and Human-Centered Design & Engineering to describe the Wisconsin Interventions in Team Science (WITS) framework, a process for translating established team science strategies into evidence-based interventions to bolster translational team effectiveness. To illustrate our use of WITS, we describe how University of Wisconsin’s Institute for Clinical and Translational Research translated the existing Collaboration Planning framework into a robust, scalable, replicable intervention. We conclude with recommendations for future SciTS research to refine and test the framework.

## Introduction

Achieving the clinical, public health, economic, and policy benefits of translational research requires collaboration among experts across biomedical, clinical, and behavioral science, as well as population health and health policy [1,2]. Accordingly, high-functioning translational teams are critical to the success of translational research [1,3–5]. Translational teams are defined as those “composed of diverse members who interact, adapt and evolve using established norms and defined roles to address a shared translational objective” [6] and often are conceptualized as a hybrid of academic research and product development teams [1]. Additional characteristics of translational teams such as evolving team membership, geographic dispersion, the diversity of disciplinary representation necessary for translational research, and the frequent inclusion of patient advocacy groups and community partners contribute to the challenges of building high-functioning teams. Moreover, these teams may represent one of several in a multiteam system, a contextual factor that may contribute to goal misalignment across these teams.

These diverse team members, and teams within teams, add both richness and complexity to team processes, and subsequently, to achieving translational team outputs, outcomes, and benefits [7]. Developing strong team processes, however, is neither quick nor easy. In general, translational researchers receive little training or support to build high-functioning teams, despite the Science of Team Science (SciTS) evidence base that has identified characteristics of such teams and their approach to teamwork. For example, engaging in multiple coordinative behaviors, developing flexible, transparent communication systems, and establishing conflict management policies increase team functioning [8–11]. Despite substantial research into collaborative science and the documentation of promising approaches to collaboration, this evidence base of effective team processes has rarely been translated into *accessible, active,* and *actionable* interventions aimed specifically at increasing translational team effectiveness. Translational teams generally lack access to effective ways to enhance team functioning due to a lack of established interventions for teams that have been rigorously tested and evaluated. Widespread availability and adoption of evidence-based interventions could help translational teams reliably improve their team processes (e.g., shared understanding of goals or coordination mechanisms), scientific outputs (e.g., peer-reviewed publications or other forms of scientific dissemination), outcomes (e.g., new knowledge, drug targets, or prototypes), and ultimately, translational science clinical, public health, economic, and policy benefits (e.g., clinical guidelines, health promotion programs, policies) [2].

For translational teams to achieve these short- and long-term goals, however, team members must be equipped with the skills, knowledge, attitudes, behaviors, and institutional support to do so effectively [6,12,13]. Delivery of team science trainings, workshops, and courses to help translational teams improve their approach to collaboration has grown in recent years, in part because some grant mechanisms, such as the CTSA Request for Applications, require this component [14]. Despite this increase, the evidence base supporting the impact of these activities on translational team outputs, outcomes, and benefits is sparse. As both the prevalence of team science initiatives and the recognition that translational team collaboration is critical to improving population health increase, so does the need to establish the translational team science intervention evidence base. In this paper, we apply lessons from Implementation Science and Human-Centered Design & Engineering to propose a rigorous process for building the evidence base for interventions designed to enhance translational team effectiveness. Building the evidence base for effective translational team interventions will enhance the effectiveness of collaborative work, the stewardship of Clinical and Translational Research (CTR) resources, and the longer-term impact to population health.

### Translating Empirically Informed Strategies into Evidence-Based Interventions

**Fig. 1.**
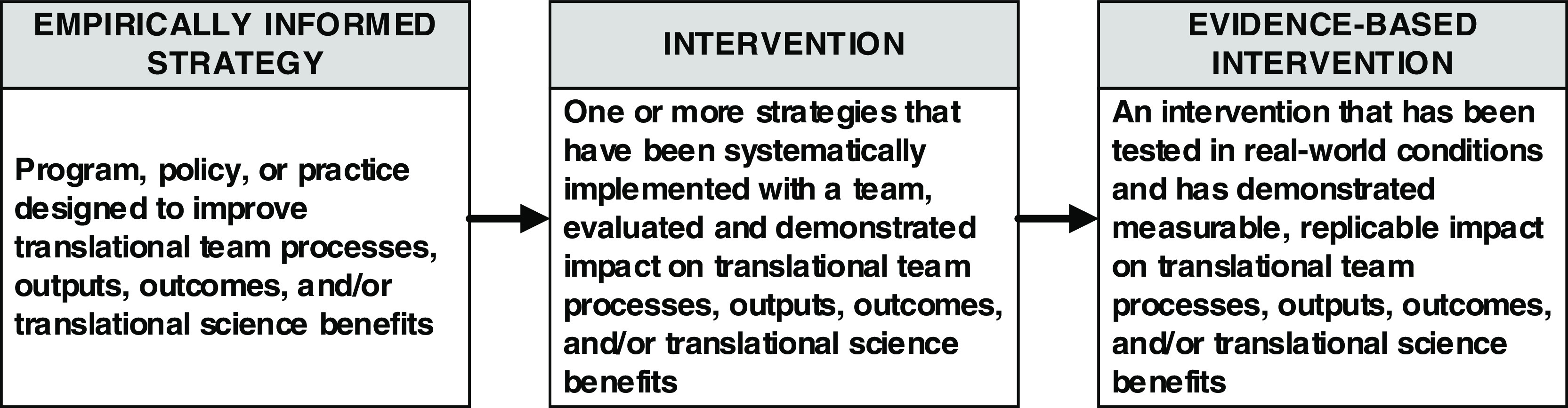
From Empirically-informed Strategies to Evidence-based Interventions.

The terms *strategy* and *intervention* are used frequently in the SciTS literature but rarely defined or contextualized. For example, the National Academies report, enhancing the effectiveness of team science, emphasizes the implementation of “actions and interventions that foster positive team processes” as a promising route to enhancing team effectiveness [7]. However, despite widespread utilization of the term intervention in the report, detailed descriptions of “interventions” were notably missing, as was any consideration of how those interventions are developed and disseminated to teams. Here, we propose a model for how existing *strategies* to promote team science success can be used to grow the evidence base for translational team science *interventions* (Fig. 1) [15,16]. This proposed typology is informed by intervention studies conducted in both translational and team effectiveness research. We define a translational team science *strategy* as a program, policy, or practice designed to improve translational team processes, outputs, outcomes, and/or translational science benefits. We broadly define a translational team science *intervention* as one strategy or a set of strategies that have been systematically implemented with a team and evaluated, with demonstrated impact. Evidence-based interventions, then, represent strategies that have been tested, with rigorous evaluation of their impact on team process in real-world settings.

### Where are the Team Science Interventions?

Studies evaluating the effectiveness of evidence-based team science interventions – particularly those implemented among translational teams – are sparse due to the inherently complex and context-specific nature of collaboration [12]. Though teams may share common challenges, they are distinct configurations of individuals with unique contextual factors impacting their functioning. These individuals have their own goals and objectives and operate within varied teams, departments, universities, disciplines, nations, and the broader culture of science. As the need for translational teams to solve complex public health and clinical challenges has grown, SciTS researchers have introduced a limited number of interventions to enhance near- and intermediate-term markers (e.g., team satisfaction and team communication) of translational team success across CTSA programs. Examples of these interventions include the Toolbox Dialogue [17], Collaboration Success Wizard [18], and TeamMAPPS [19]. This body of work has demonstrated that conducting interventions across diverse translational science settings is feasible, if challenging, and that teams receiving these interventions have demonstrated improvement in various aspects of team functioning. However, the small number of studies, their small sample sizes, overrepresentation of case and observational rather than experimental designs, and limited systematic guidance for intervention adaptation across settings hinder their capacity to scale and, consequently, their utility. These factors highlight an opportunity for the SciTS field to engage in the development of interventions to bolster translational teams’ collaborative capacity.

### From Strategy to Intervention: The Wisconsin Interventions for Team Science (WITS) Framework

One overarching challenge for the SciTS field is the lack of rigorous approaches to test and evaluate team-based interventions. Gold standard testing methodologies such as randomized controlled trials (RCTs), so popular in other fields, can be difficult to execute and, not least of all, to pay for given federal funding agencies’ lack of funding for SciTS research. The field lacks methods to design, build, evaluate, disseminate, and implement interventions in a way that allows us to claim evidence-based effectiveness. In this section, we propose an approach to developing team science interventions, drawing upon research from Human Centered Design & Engineering (HCDE) and Implementation Science. We envision this framework being used by SciTS researchers and team science facilitators who are interested in translating their research findings into empirically informed strategies and then into evidence-based interventions that enhance team functioning and productivity outcomes for translational teams.

**Fig. 2.**
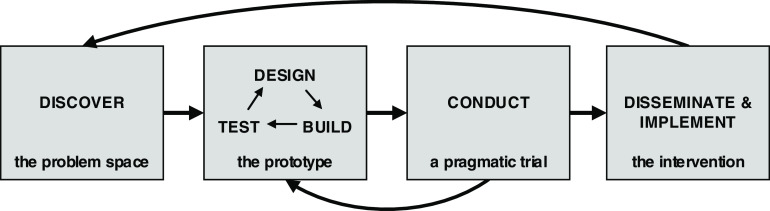
Wisconsin Interventions for Team Science Framework: A Four-Phase Approach to Team Science Intervention Development.

The current state of team science intervention development is such that initiative directors and translational scientists who work in or lead team science initiatives have been left to identify ways to implement strategies and/or interventions in their local contexts, without any guidance as to how they can do so with fidelity (i.e., as they were intended to be implemented) or how usable the interventions will be in their teams. The Wisconsin Interventions for Team Science (WITS) Framework is a dynamic framework to guide the iterative processes involved in translating one or more team science strategies into an intervention (Fig. 2). Informed by the Discover, Design/Build, and Test (DDBT) framework [20] and the Diffusion-Dissemination-Implementation Continuum [21], the proposed framework emphasizes the integration of concepts from Implementation Science and HCDE, two fields with mature, rigorous approaches to developing and scaling interventions. Both disciplines focus on the importance of “designing for dissemination” and prioritizing the co-creation of knowledge with the input and understanding of the needs and characteristics of priority stakeholders [22, 23]. While this approach to intervention development is not new, it has not previously been applied to SciTS in a systematic way.

The WITS framework is comprised of four phases: (1) Discover; (2) Design, Build, and Test; (3) Conduct a pragmatic trial; and (4) Disseminate and Implement. In the following sections, we describe each stage of the WITS framework and then illustrate its use with case examples from our own work with Collaboration Planning, a popular team science framework. At the University of Wisconsin-Madison Institute for Clinical and Translational Research (UW-ICTR), we adapted this strategic framework and delivered its combination of activities as an intervention [24]. UW-ICTR’s ultimate goal is to disseminate and implement Collaboration Planning as an *evidence-based intervention* that can be used across the CTSA consortium and beyond. For each WITS phase, we describe the goal, as well as proposed activities and process indicators – direct products of each phase’s activities – to evaluate whether the intervention is being developed as planned. Table 1 contains a summary of the goals, activities, and process indicators for each phase. To give a better sense for how the framework might play out in developing an intervention, here we also briefly present our development process for adapting the Hall, Vogel, and Crowston Collaboration Planning framework [25] into an intervention (Phase 1 and 2) and how we anticipate translating that into an evidence-based intervention (Phase 3 and 4) [24].

**Table 1.** Summary of Wisconsin interventions for team science framework phases, goals, activities, and process indicators

Activities	Process indicators
**Phase 1: Discover**	
Goals: Understand the problem space; identify the challenge translational teams are experiencing; identify end users (i.e., the target audience for the intervention); describe context in which users operate	
• Identify and assess the characteristics, needs, and challenges experienced by the end users • Identify potential strategies to meet the identified translational team needs.	• Translational team challenge identified • Intervention outcomes and endpoints defined • Strategies for translation enumerated • End users and stakeholders identified • Context described
**Phase 2: Design, build, and test**	
Goal: Design, build, and test the intervention	
• Design the intervention • Build the intervention prototype • Test the intervention prototype	• Plan for intervention developed • Intervention components described • Intervention form established • Dissemination plan drafted • Training plan for intervention delivery established • Testing plan developed • Working prototype created • Testing plan implemented
**Phase 3: Conduct a pragmatic trial**	
Goal: Test the intervention rigorously	
• Operationalize the defined outcomes and endpoints • Implement previously planned pragmatic trial • Recruit additional, representative teams • Collect, manage, and analyze evaluation data	• Defined endpoints successfully met • Pragmatic trial implemented as planned • Number of teams recruited • Data collected, managed, and analyzed
**Phase 4: Disseminate and Implement**	
Goal: Disseminate and scale up the evidence-based intervention	
• Review, revise, and implement the dissemination plan • Engage local Dissemination and Implementation experts • Collect, manage, and analyze data to monitor effectiveness	• Number of teams engaged for intervention dissemination • Settings/types of organizations, academic departments, and/or teams represented by those engaged for intervention • Adaptations needed for new contexts identified

### Phase 1: Discover

#### Goal

The goal of the Discover phase is to understand the problem space, including the challenge translational teams are experiencing that the intervention should solve, the end users (i.e., the target audience for the intervention), and the context in which those end users operate.

#### Activities

This phase consists of two key activities: (1) identify and assess the characteristics, needs, and challenges experienced by the end users and (2) identify potential strategies to meet the identified translational team needs.

##### Identify and assess the characteristics, needs, and challenges

Assessing the needs of translational scientists and teams can be achieved using quantitative and qualitative data collected through needs assessments or structured observation [26]. An important component of this phase is convening a group of stakeholders to define and understand the problem space of interest, taking the ecosystem perspective-discovering needs of the priority population (individuals), the teams in which they operate, and the institution/institutional multiteam system that may influence the implementation of the intervention. It is also important in this process to understand what the team’s desired state is; in other words, what will success for this intervention look like, and for whom? Assessing the needs of the end users increases the likelihood of feasibility and usability of the intervention by ensuring it is designed to meet the needs of the stakeholders. Intervention developers do not need to start from scratch in understanding the needs of translational teams and the context in which they operate. For example, the Clinical and Translational Science Personas project has described key aspects of those working in CTR [27]. The SciTS literature, too, has described key influences on science team functioning, such as attitudes toward collaboration, leadership, trust, and incentives to collaborate, that can serve as background for the needs assessment [8,9,12]. Part of understanding the needs of the translational team is understanding the contextual and environmental conditions that support effective teams, while simultaneously illuminating gaps and barriers in those conditions to implementation [8]. In other words, the needs assessment is critical to understanding why the challenge currently exists, who will receive the intervention, and in what context.

##### Identify potential strategies to meet the identified translational team needs

Once the need has been identified, documented, and understood, the next activity in the Discover phase is to identify potential strategies that can be implemented to address the challenge the translational team is experiencing. The SciTS literature is one source for these strategies, as many teams and SciTS researchers have written about ways teams have addressed team-based barriers. The second source of strategies is teams themselves. Many science teams have published anecdotal accounts of their team science challenges and tools or approaches they developed to address those, often in their domain-specific literature (e.g., teams working in cancer research often publish these accounts in cancer-focused journals [28, 29]). Those accounts can be rich sources of both contextual information and ideas for mitigating challenges.

#### Process indicators

Success for the Discover phase includes co-created knowledge and identification of: the teams’ challenge, the desired outcome of the intervention, the context in which the end users operate, and strategies the team is considering translating into an evidence-based intervention. Stakeholder engagement in this phase is critical, and we encourage intervention developers to identify representative end users, as well as institutional leadership who may be impacted by the intervention, to engage in this co-creation of the needs assessment. Brief interviews with these stakeholders can ensure a full picture of the need. There are many ways to document the output from the Discover phase, including an asset map, a co-created action plan for designing the intervention, a narrative description of the challenge and population, or a description of how the developers envision the strategies working to address the challenge.

#### Collaboration Planning at UW-ICTR

The challenge that we identified was the difficulties some UW-ICTR pilot teams experienced in developing strong team processes while launching their projects. Teams were often newly formed, with limited resources for supporting collaborative work. The UW-ICTR team science team worked closely with our pilot program administrators and Workforce Development team to understand the challenges experienced by the teams and those experienced by the pilot program itself in helping the pilot teams successfully execute their projects. We already had a strong understanding of our institutional context, because we were supporting other translational teams, so our primary discovery work was to understand what strategies might be available to help address our challenge. We identified Collaboration Planning as a relatively lightweight (i.e., not needing substantial time investments), accessible framework that we believed could be adapted to an empirically informed strategy and then translated into an evidence-informed intervention that impacted team processes and, eventually, translational team outputs, outcomes, and/or translational science benefits.

### Phase 2: Design, Build, and Test (DBT)

#### Goal

The goal of the second phase is to design, build, and test the intervention, drawing upon the outcomes of the Discover phase to create a prototype.

#### Activities

In this second phase, we draw upon HCDE process and Implementation Science to “design for dissemination” by synthesizing findings from Phase 1 to iteratively design and build a prototype intervention and pilot test the prototype such that it is as responsive as possible to the needs of the target audience. This approach facilitates continuous improvement to the prototype design and implementation approach. In addition to the Design, Build, and Test steps being iterative, it is possible that these steps will reveal the need to return to the Discover phase to learn additional contextual information.

##### Design

The key activity in the Design step is the development of a plan for the intervention, including: (1) the components of the intervention, including how the identified strategies will be adapted; (2) the form the intervention will take (e.g., workshop, facilitated discussion, online assessment, consultation); (3) how success of the intervention will be measured; (4) how the intervention will be disseminated, including how the intervention will reach the adopters (those responsible for bringing the intervention to their institution), the implementers (those delivering the intervention), and the end users; (5) how will implementers be trained in delivering the intervention; and (6) how the intervention will be tested, both in the pilot testing and pragmatic testing in Phase 3. (For additional guidance on intervention design considerations, please see Rolland *et al.* [30]. Decisions on all of these design elements should be dictated by the assessment of the team’s needs and their context conducted in the Discover phase. A key part of designing the intervention is developing outcome measures and an evaluation framework for assessing its effectiveness, as well as creating a dissemination plan that lays out how the intervention will be shared with teams at CTSA hubs and beyond.

##### Build

Building the intervention prototype is the next step in the DBT phase. Depending on the format of the intervention, existing activities, measures or scales may be adapted or used in the prototype. Key to success in this phase is a working prototype that meets the needs of the adopters, implementers, and end users, as well as a detailed plan for Phase 3, the pragmatic trial. That trial design should be informed by the measures of success defined during the Design activities. In addition, as part of the iterative process of DBT, subsequent build phases should be informed by efficacy data generated from pilot testing.

##### Test

Testing of the prototype can be done with the stakeholders engaged in the Discover phase, or additional teams whose needs and contexts are similar to the targeted end users can be recruited for testing. The approach to testing should be dictated by the defined outcomes and desired post-intervention state, informed by the metrics of success previously defined. As noted above, this is an area ripe for innovation for SciTS researchers.

#### Process indicators

Success for Phase 2 is an intervention that is ready to be tested more broadly, in real-world conditions through a pragmatic trial. Potential process indicators for design activities might include a plan for the intervention, a description of the intervention components and form, and plans for dissemination, training, and testing. Indicators for the build phase include a working prototype, and the test phase indicators might include the completion of the test plan. It should be noted that many cycles of Design/Build/Test may be required to get to the point where the intervention is ready to move to pragmatic testing. As discussed above, it may be the case that Phase 2 actually leads back to an additional engagement with Phase 1 if the development team feels like key pieces of information about the team, its context, or the chosen strategies are missing or need to be expanded upon.

#### Collaboration Planning at UW-ICTR

In this phase, we adapted the Collaboration Planning framework to meet the needs of our pilot teams, drawing upon our understanding of SciTS research, our own experience working with translational teams, and our success metrics in mind. We wanted an accessible, active, actionable intervention that could be delivered in around 90 minutes, required no additional pre-session training for participants, included as many members of the team as possible actively engaged in defining their team processes, and provided the teams with a roadmap for their collaborative work. At this point, we had not yet developed a plan for how the intervention could be disseminated more broadly but were simply trying to design an intervention that would work for our local teams in our local context. Our evaluation plan was also quite simple and focused primarily on team engagement and perceived value of the process.

The prototype intervention we built consisted of a worksheet of questions in each of the 10 focal areas laid out in the original Collaboration Planning framework. The intervention was designed to be led by a facilitator [BR] leading the team through the questions, with a team deliverable of a written Collaboration Plan to be submitted to the pilot program administrators with the team’s first quarterly report. Also part of the prototype was the invitation letter we sent to teams inviting them to participate, as well as our evaluation plan. Subsequent iterations, most recently delivered Fall 2020, included a registration survey that asked additional questions about the team as we sought to customize or target the intervention to the type of team (e.g., community-engaged, first-time PI, junior–senior partnership), a pre-session survey to understand their comfort level with the aspects of team processes, and a post-session survey to assess their perceived value of the session, which areas they anticipated using the most, and any suggestions for improving the process.

As we began delivering the intervention and testing it with our pilot teams, a member of the UW-ICTR evaluation team observed each session, identifying language in the intervention that did not resonate with the team members, that was confusing, or did not result in the intended discussion. Results of this pilot test and some of the challenges we observed are described elsewhere [24], and the feedback from our observations and surveys of participants has been integrated into subsequent versions of the intervention, which are then tested with new teams. We are currently testing version 6 of the worksheet, using what we learn in each session and each round of testing to improve our approach. This focus on continuous improvement is key for making the testing activities as efficient as possible.

### Phase 3: Conduct a Pragmatic Trial

#### Goal

The goal of Phase 3 is to test the intervention rigorously using the approach known as a pragmatic trial. While RCTs are generally both unrealistic and expensive for team-based interventions, a pragmatic trial can be a simpler, more effective, and generalizable approach for team science intervention testing that still provides rigor. Pragmatic trials are often used in clinical settings to inform decisions about practice and to accelerate adoption of evidence-based interventions. They are designed to evaluate effectiveness of an intervention in a real-world setting before releasing the intervention more broadly [31,32]. While the previous DBT phase helps assess if the intervention *can* work (efficacy), pragmatic trials determine whether the intervention works across team science settings (effectiveness) in a small group of representative users. In this phase, participants are not randomly selected; rather they represent members of one or more translational teams. Because of the careful attention and time investment, as well as the engagement of stakeholders in the Discover and DBT phases, it is expected that the pragmatic trial will progress rapidly to the next phase, Disseminate and Implement, when the intervention is available more broadly.

#### Activities

Core activities of the pragmatic trial include the operationalization of the defined outcomes and endpoints, implementation of the pragmatic trial plan previously developed, as well as the recruitment of additional, representative teams. The intervention must be packaged in a way that implementers are able to deliver the intervention with fidelity, even if the original developers are not the ones delivering the intervention. This phase also involves the collection, management, and analysis of evaluation data.

#### Process indicators

The outcome of a successful pragmatic trial, that is, one that has successfully met its endpoints, is an evidence-based intervention. Success for the pragmatic trial must be defined in advance to ensure that the trial is truly measuring the targeted improvements in translational team outputs, outcomes, and/or translational science benefits. What these outputs, outcomes, and/or translational science benefits are depends on the objectives of the intervention, but may include, for example, improved scores on a collaboration readiness scale, increased psychological safety in the team, or a successful grant submission with a community partner. Once a successful trial has been conducted, the intervention is ready to be disseminated and implemented in additional settings. Much work remains to be done by the SciTS field in identifying short- and medium-term outputs and outcomes that indicate improved team functioning.

#### Collaboration Planning at UW-ICTR

Having now delivered the intervention to more than 25 teams at the UW, we are ready to begin the next phase, gearing up to conduct a pragmatic trial of the Collaboration Planning intervention in a variety of context, including a large UW-based research center and a public university interested in increasing the number of large center grants they receive. In anticipation of this phase, we have engaged our UW-ICTR Dissemination & Implementation Launchpad team to help us design the trial and our dissemination strategy. Ideally, we would have done this step in Phase 2 while designing this intervention, drawing upon the literature from the field of Implementation Science that posits that considering the dissemination plan earlier rather than later in intervention development leads to better outcomes [33].

In anticipation of the pragmatic trial, we have developed two additional components, a Facilitators Guide and a Facilitator Training to ensure fidelity to the intervention, and are developing a comprehensive evaluation plan designed to measure both team process outcomes (e.g., increases in trust among team members) and team outputs (e.g., papers, future grants, patents) in a consistent way across implementation of our intervention. The Facilitators Guide codifies our approach to delivering the intervention, including introductions for each of the 10 focal areas that highlight why each area is important in a way that is accessible for the teams, not requiring any prior knowledge of SciTS. In recent months, we have also developed a Facilitator Training to increase our ability to scale the intervention beyond the UW. So far, we have pilot tested that Facilitator Training with two individuals interested in delivering Collaboration Planning themselves. Finally, our evaluation plan is underway, and we are investigating which established measurement tools may be useful for assessing our outcomes of interest.

### Phase 4: Disseminate and Implement

#### Goal

The goal of the final phase, Disseminate and Implement, is to disseminate the evidence-based intervention according to the dissemination plan developed during the design step of Phase 2 and scale up the implementation of the intervention broadly. This phase is differentiated from Phases 1 and 2 based on the scale at which the intervention is implemented. Whereas Phase 3 focused on spread (i.e., replicating the intervention in alternative settings), Phase 4 encompasses both spread and scale-up (i.e., integrating support for interventions into organizational practices and infrastructure) [34].

#### Activities

The activities of Phase 4 are determined by the form and desired outcomes of the intervention, as well as the dissemination plan designed earlier. There are many ways to disseminate an intervention, including by offering a service to teams that may be interested in implementing the intervention, creating a self-guided intervention, or training a cadre of facilitators who deliver the intervention to their teams. The packaged intervention may include a facilitator’s guide or instructions on how teams can implement the intervention themselves. We highly recommend the engagement of local Dissemination and Implementation experts in this phase in order to take full advantage of the knowledge that field has developed in successfully getting interventions to those who need them. Feedback from the D&I phase should also be collected and used to improve the initial Discovery phase, as indicated in Fig. 2 by the arrow pointing back to Phase 1. In this way, the evidence base for the intervention’s effectiveness continues to build and improve.

#### Process indicators

Success for the Dissemination and Implementation phase is the generation of knowledge about how the intervention is being used and its short-, medium-, and long-term impact on translational teams at CTSA hubs and beyond, as well as the longer-term impact and achievement of translational science benefits. Again, it is critical here to have plans for evaluating the intervention “in the wild” and using lessons learned from that implementation to continuously improve the intervention. Potential indicators might include number of teams engaged for intervention dissemination, settings or types of organizations/departments/teams represented by those engaged for intervention, or adaptations needed for new contexts. Similarly, it is critical for the SciTS field that intervention developers report the results of their testing. Simply releasing the intervention without plans for continuing to evaluate it is a missed opportunity for increasing our knowledge of both intervention development and the ways that teams use interventions in real-world settings. Evaluation metrics from the field of Implementation Science can be useful in considering what continued evaluation might entail. Specifically, the RE-AIM framework has been widely used to understand the outcomes and impact of the implementation of health-based interventions [35].

#### Collaboration Planning at UW-ICTR

The two biggest challenges we anticipate with disseminating and implementing this intervention more broadly are fidelity and scaling up. We have addressed both challenges, to some extent, through the development of a Facilitators Guide and Facilitators Training. The Facilitators Guide includes specific language to use in introducing the session and engaging with participants, increasing both its feasibility and usability by not requiring the facilitator to become an expert in the SciTS. By training facilitators, the intervention can be delivered at multiple institutions, both allowing us to scale the intervention in a manageable way and providing additional opportunities for evaluating its effectiveness in a variety of settings. An additional challenge will be continued data collection on the effectiveness of the intervention once it has been widely disseminated. We hope to prevail upon teams to share their output and outcome data back to UW-ICTR, so we can continue to track these metrics.

### Summary of Framework and Next Steps

Based on our development of the Collaboration Planning intervention, and our training in and experience with Human-Centered Design & Engineering, Implementation Science, and the SciTS, we created the WITS framework as a way to echo the question posed by Peek and colleagues, “How do we continue to adapt and spread what we learn in practice?” [36] This initial description of the proposed framework is designed to equip stakeholders engaged in translational team science with a practical way of conceptualizing the iterative process of intervention development: discovering the needs and context of stakeholders; designing, building, and testing the intervention; conducting a pragmatic test of the intervention; and widespread dissemination and implementation. We propose that this process will provide an opportunity for the SciTS field to engage in rigorous and transparent translation of the many existing team science strategies into evidence-based interventions with proven impact on translational team outputs, outcomes, and/or translational science benefits. Furthermore, by publishing evaluation plans, we can begin to develop a set of metrics that can be used to examine effectiveness.

Although the WITS framework holds promise for enhancing translational team functioning, there are some notable limitations to our work. First, our evaluation framework proposes performance indicators as a way to monitor the intervention design and delivery. These indicators are not necessarily representative of the longer-term translational team outcomes, impacts, and benefits conferred by this intervention. In addition, a challenge not unique to team science is the need to adapt interventions to accommodate cultural norms, new target populations, and new settings, while maintaining intervention fidelity [37]. As we continue to implement, disseminate, and evaluate WITS, we plan to evaluate the longer-term impacts of our work on team, institutional, and scientific outcomes, as well as adaptations needed as we increase spread and scale-up of our work.

Next steps for the WITS framework include continuing to flesh out both the activities and the metrics for each phase. We hypothesize that, even with disparate intervention outcomes and approaches, there will be common metrics that can be developed for each of the phases and activities of the process. We have begun this work in Rolland *et al.* [30], but there is still much to be done in honing and testing these metrics. We also plan to continue investigating methods of testing interventions that are easily applied to team-based intervention and also increase the rigor and reproducibility of evaluation results. Finally, we hope to create tools (e.g., worksheets or checklists) that aid intervention developers in following our approach. Our hope is that other SciTS and team researchers will use this framework as a starting point for their own investigations of providing evidence-based interventions and other tools to translational teams.

## Conclusion

As an emerging discipline, the SciTS field currently lacks reliable, valid tools, and methodological guidance to advance the translation of empirically informed strategies to experimentally tested, evidence-based interventions. As we seek to support translational teams in their important efforts to address complex health challenges, there is an urgent need to reassess traditional approaches to developing the evidence base for informing the design, implementation, and dissemination of translational team interventions. We need rigorous approaches that produce feasible and usable interventions that we can confidently claim impact translational team outputs, outcomes, and/or translational science benefits.

The proposed WITS framework outlines a systematic approach to intervention development that SciTS researchers can use to translate their findings into accessible, active, and actionable evidence-based interventions. Our goal is to provide a framework for the development and field-wide adoption of rigorous methods to assess the efficacy and effectiveness of translational team interventions, so they can be systematically implemented and integrated in formal team science initiatives across clinical and translational research networks.
